# Genetic and phenotypic characteristics of four Chinese families with fundus albipunctatus

**DOI:** 10.1038/srep46285

**Published:** 2017-04-10

**Authors:** Guoxing Yang, Zhiqiang Liu, Shipeng Xie, Chengquan Li, Lina Lv, Minglian Zhang, Jialiang Zhao

**Affiliations:** 1 Department of Opthalmology, Hebei Provincial Eye Hospital, Hebei, China; 2 Hebei Provincial Key laboratory of ophthalmology, Hebei, China; 3 Department of Opthalmology, Peking Union Medical College Hospital, Chinese Academy of Medical Sciences & Peking Union Medical College, Beijing, China

## Abstract

Fundus albipunctatus (FA) is a rare autosomal recessive form of stationary night blindness characterized by the presence of white or white-yellow dots in the perimacular area and the periphery of the retina, with or without macular involvement. In this study, we examined four Chinese families with FA. Patients were given complete ophthalmic examinations, and blood samples were collected for DNA extraction. Three genes, *RDH5, RLBP1* and *RPE65,* were screened by direct sequencing. Mutations in *RDH5* were identified in three families and mutations in *RPE65* were identified in one family. This is the second reported case of FA caused by mutations in *RPE65*.

Fundus albipunctatus (FA) is a rare autosomal recessive form of stationary night blindness characterized by the presence of white or white-yellow dots in the perimacular area and the periphery of the retina, with or without macular involvement. This disease was first described by Lauber in 1910 who distinguished it from an ophthalmoscopically similar disorder called retinitis punctata albescens^1^.

Retinol dehydrogenase 5 (*RDH5*) mutations that cause FA were reported by Yamamoto in 1999^2^. *RDH5* is expressed predominantly in retinal pigmented epithelium (RPE) and encodes for 11-cis retinol dehydrogenase. This retinol dehydrogenase functions to catalyze the final step in the biosynthesis of 11-cis retinaldehyde, which is the universal chromophore of visual pigments^3^.

*RPE65* mutations have been associated with Leber’s congenital amaurosis type 2 (LCA2) and retinitis pigmentosa^4^,^5^. The RPE65 protein is expressed in the RPE and is involved in the conversion of all-trans retinol to 11-cis retinal in the retinoid cycle. It plays important roles in the production of 11-cis retinal and in visual pigment regeneration^6^. Several studies demonstrate that RDH5 forms a complex with RPE65 in RPE^7^,^8^,^9^,^10^.

Mutations in three key retinoid cycle enzyme: LRAT (lecithin retinol acyltransferase), RDHs (retinol dehydrogenases) and RPE65 and RLBP1 (retinaldehyde binding protein 1) genes were associated with the appearance of white-yellow dots on fundus examination^11^,^12^,^13^,^14^,^15^.

In this study, we described the clinical features and molecular genetic results in four patients affected with FA.

## Results

### Clinical findings

**Figure 1:**
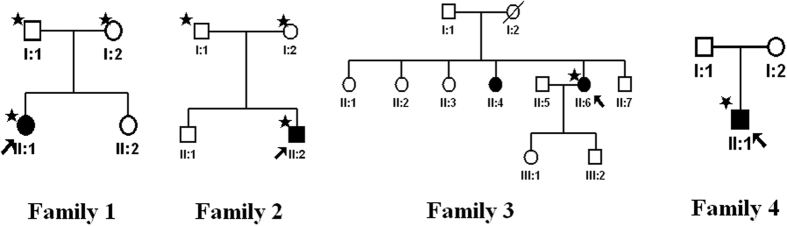
Pedigrees of four families with fundus albipunctatus. Asterisks represent the participants and the proband is denoted by an arrow.

**Figure 2:**
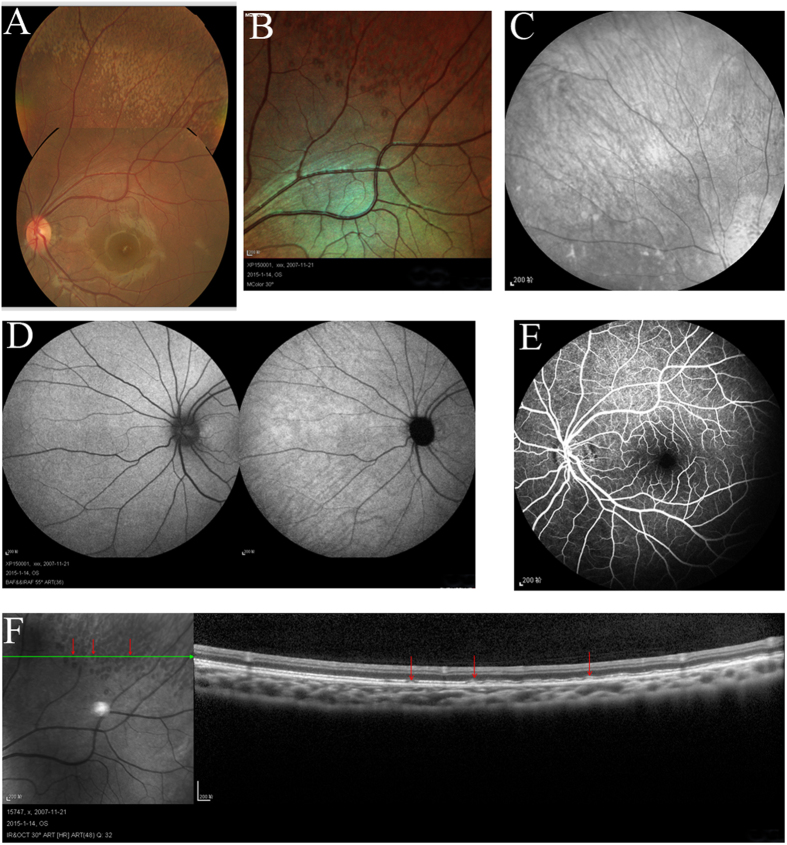
Fundus imaging of II:1 in family 1. Fundus photograph (**A**) and multicolor (**B**) showed lots of white-yellow dots in the periphery of the retina. IR (**C**) showed the dots in areas corresponding to fundus photograph. FAF (**D**) and FFA (**E**) showed the normal image. OCT (**F**) showed the hyperreflective lesions (arrows) in areas corresponding to dots (arrows) in the periphery of the retina. Abbreviations: IR. infrared reflectance; FAF. fundus autofluorescence; FFA. fundus fluorescein angiography; OCT. optical coherence tomography.

**Figure 3:**
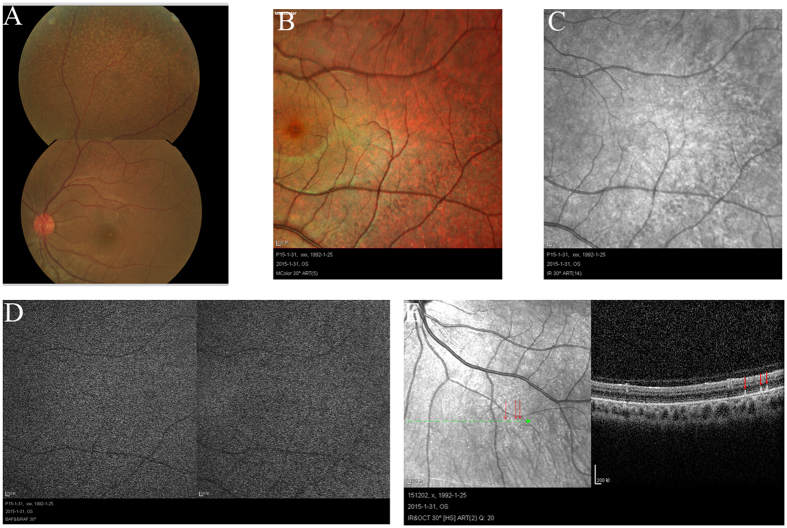
Fundus imaging of II:2 in family 2. Fundus photograph (**A**) and multicolor (**B**) showed lots of white-yellow dots in the periphery of the retina. IR (**C**) showed the dots in areas corresponding to fundus photograph. FAF (**D**) showed the normal image. OCT (**E**) showed the hyperreflective lesions (arrows) in areas corresponding to dots (arrows) in the periphery of the retina. Abbreviations: IR. infrared reflectance; FAF. fundus autofluorescence; OCT. optical coherence tomography.

**Figure 4:**
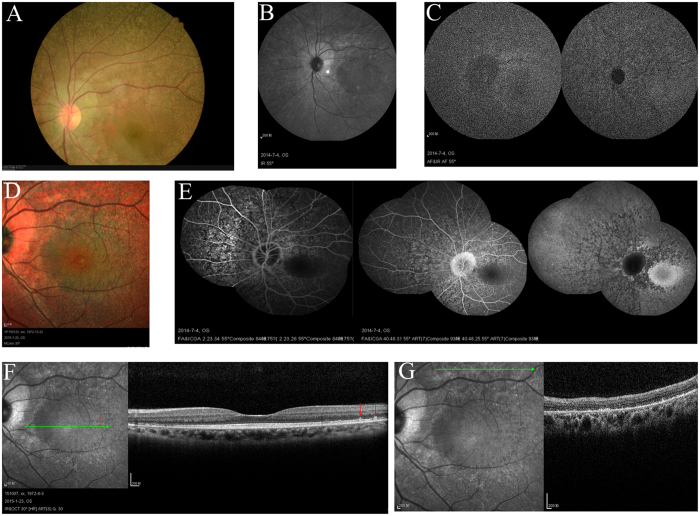
Fundus imaging of II:6 in family 3. Fundus photograph (**A**) and multicolor (**D**) showed lots of white-yellow dots in the perimacular area and the periphery of the retina. IR (**B**) showed the dots in areas corresponding to fundus photograph. FAF (**C**) showed the normal image. FFA/ICGA (**E**) showed the RPE lesions in the retina with preservation of the macular area. OCT (**F**,**G**) showed the hyperreflective lesions (arrows) in areas corresponding to dots (arrows) in the perimacular area and atrophy of IS/OS-RPE in the periphery of the retina. Abbreviations: IR. infrared reflectance; FAF. fundus autofluorescence; FFA/ICGA. fundus fluorescein angiography/indocyanine green angiography; OCT. optical coherence tomography; IS/OS.inner-segment/outer-segment; RPE. retinal pigment epithelium.

**Figure 5:**
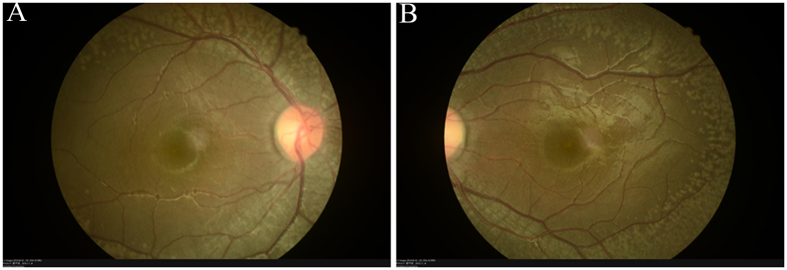
Fundus imaging of I:1 in family 4. Fundus photograph (**A**,**B**) showed lots of white-yellow dots in the periphery of the retina.

**Figure 6:**
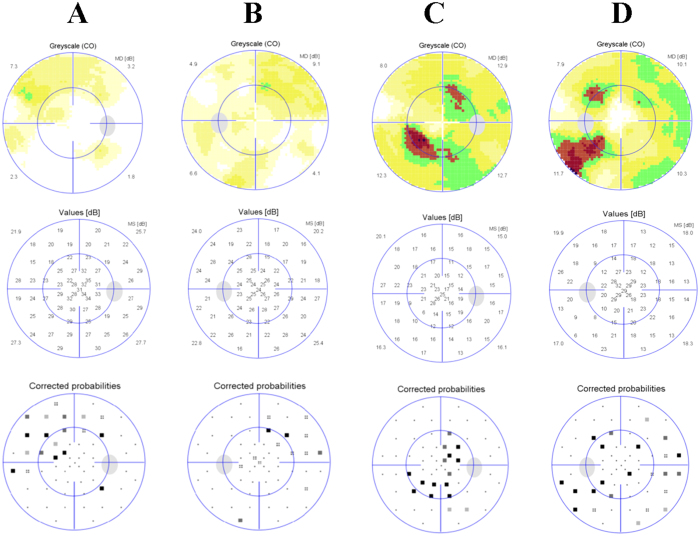
Visual field of II:1 in family 1 and II:6 in family 3. Visual field of II:1 in family 1 showed mild reduction of sensitivity in the central visual field (**A**,**B**). Visual field of II:1 in family 3 showed moderate reduction of sensitivity in the central visual field (**C**,**D**).

**Figure 7:**
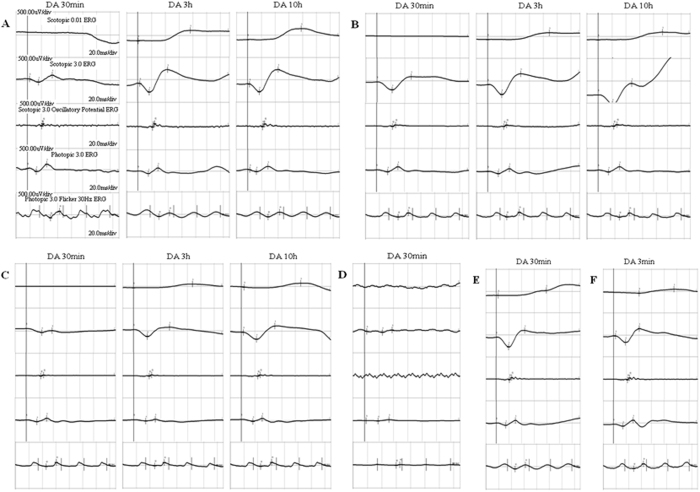
Full field electroretinography (ffERG). (**A**) II:1 in family 1. (**B**) II:2 in family 2. (**C**) II:6 in family 3. (**D**) II:1 in family 4. (**E** and **F**): Normal control. ffERG showed no rod responses. After prolonged dark adaption (3 h, 10 h), the rod responses almost recovered to normal levels.

The patients II-1 in family 1, II-2 in family 2, II-6 in family 3 and II-1 in family 4 showed typical FA on examination. (Figs 1, 2, 3, 4, 5, Table 1). All patients showed white-yellow dots scattered in the periphery of the retina. In addition, II-6 in family 3 showed white-yellow dots scattered in the perimacular area. All affected patients experienced poor night vision at an early age. Infrared reflectance (IR) images showed the dots were in areas corresponding to fundus photograph and fundus autofluorescence (FAF) images are normal. Retinal venous phase images of fundus fluorescein angiography (FFA) of the II-6 in family 3 showed extensive mottled staining hyperfluorescence and crack-like hypofluorescence with preservation of the macular area (Fig. 4). Late phase images of indocyanine green angiography (ICGA) of the II-6 in family 3 showed extensive cord-like hypofluorescence with preservation of the macular area and ring-like hyperfluorescence in the macular area. The OCT of the patients showed a lot of hyper-reflective lesions corresponding to white-yellow dots on fundus. Hyper-reflective lesions extend from the RPE to the external limiting membrane (Figs 2, 3, 4). Visual field of patients showed reduction of sensitivity in the central visual field (Fig. 6). Full field electroretinography (ffERG) showed no rod responses according to the ERG standards of the International Society for Clinical Electrophysiology of Vision (ISCEV, 2008 Version). After prolonged dark adaption (3 h, 10 h), the rod responses almost recovered to normal levels (Fig. 7, Table 2). Physical examinations excluded systemic disorders in all patients.

### Mutation analysis

**Figure 8:**
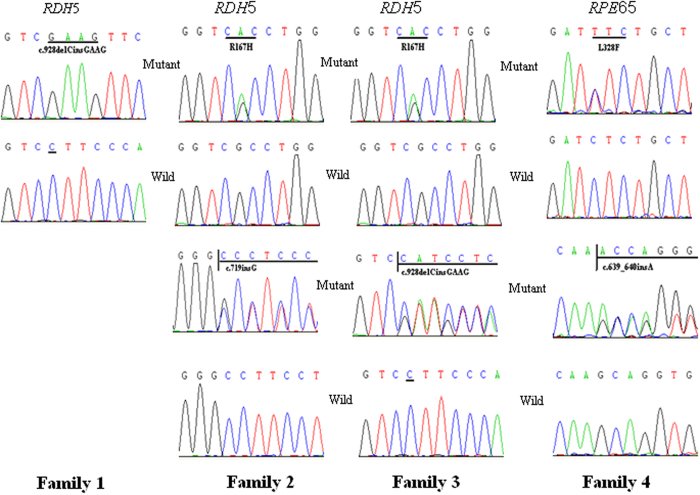
DNA sequences of *RDH5* and *RPE65* in affected individuals and controls. A homozygous c.928delCinsGAAG (Leu310 to GluVal) mutation of *RDH5* in family 1. Two heterozygous mutations of *RDH5*, c.500 G > A (p.Arg167His) and c.719ins G (p.Ala240Glyfs17), in family 2. Two heterozygous mutations of *RDH5*, c.928delCinsGAAG (Leu310 to GluVal) and c.500 G > A (p.Arg167His), in family 3. Two heterozygous mutations of *RPE65*, c.639_640insA and c.982 C > T (L328F), in family 4.

Mutations of *RDH5* were identified in three families, including a homozygous c.928delCinsGAAG (Leu310 to GluVal) mutation in family 1, heterozygous c.500 G > A (p.Arg167His) and c.719insG (p.Ala240Glyfs17) mutations in family 2 and heterozygous c.928delCinsGAAG (Leu310 to GluVal) and c.500 G > A (p.Arg167His) mutations in family 3. In family 1, mother and father both carried the c.928delCinsGAAG mutation. In family 2, the father carried c.500 G > A mutation and the mother carried the c.719insG mutation respectively. Mutations of *RPE65* were identified in family 4, including heterozygous c.639_640insA and L328F mutations (Fig. 8). The c.639_640insA was predicted to lead to premature stop codons (p.A214Sfs20) and cause a loss of function. The p.L328F change is predicted to damage the function of RPE65 when analysed using SIFT (0.998) and Polyphen-2 (0.01) websites.

## Discussion

In this study, four patients were ascertained who exhibited typical FA. Four patients in four different families include three children and one middle-aged woman. They all suffered from night blindness from birth. We identified three disease-causing mutations in RDH5 in three unrelated families and two potential disease-causing mutations in RPE65 in one family with FA. In previous reports, about 50 different mutations in *RDH5* associated with FA have been identified (data from Human Gene Mutation Database^16^). The FA in Asian, Israeli and Pakistani patients share common mutations in *RDH5*, suggesting that most mutations are inherited from carriers in the population instead of being a-*de novo* mutations^13^,^17^,^18^. One study in 2011 reported *RPE65* mutations caused FA in an 18-year-old woman^11^.

The retinoid (visual) cycle is an enzyme pathway that occurs to regenerate the visual chromophore following light exposure. The RDH5 and RPE65 encode two key catalytic enzymes participating in the retinoid (visual) cycle. A study by Driessen and colleagues demonstrated that *Rdh5*^−/−^ mice show an accumulation of cis-retinols and cis-retinyl esters^19^. The accumulation of cis-retinoids may be responsible for the pathology of FA. Other enzymes in the RPE may compensate for the isomerohydrolase activity. A study confirmed that RDH10 can partially compensate for the loss of RDH5 function^20^. This may explain why the ffERG response can recover after dark adaption in patients with FA and why FA has relatively mild symptoms compared with RP.

The white dots in the fundus varied with age and genotype. Fundus autofluorescence (FAF) was performed in three patients. Two patients showed subnormal FAF. Autofluorescence in the retinal pigment epithelium represented the accumulation level of lipofuscin. A2E is a major component of lipofuscin. The mutations in RDH5 and RPE65 affected retinoid metabolism in the visual cycle and the production of A2E in the RPE. Spectral domain-OCT in the patients showed a lot of hyper-reflective lesions corresponding to white-yellow dots on fundus. Hyper-reflective lesions extend from the RPE to the external limiting membrane and distributed in the periphery except for the macular area. After prolonged dark adaption (>3 h), the rod responses almost recovered to normal levels. The reason may be explained by an alternative compensation mechanism in the visual cycle. Further work is needed to understand the dark adaptation in fundus albipunctatus.

Gene therapy for *RPE65*-related Leber congenital amaurosis has been shown to be a successful and innovative technology in translational research^21^,^22^,^23^. It has become a research hotspot. Mutations of *RPE65* can cause different phenotypes, but the mechanisms are still not clear. Our study provides valuable material and clues for further research about the biological functions of *RPE65* and the pathogenesis of FA.

## Methods

### Patients and clinical data

The four families enrolled in this study were from Hebei province, China. Clinical examination, peripheral blood collection and DNA extraction were performed at the Department of Ophthalmology at Hebei Ophthalmic Hospital. This study followed the tenets of the Declaration of Helsinki, and was approved by the Ethics Committee of Hebei Provincial Ophthalmic Hospital. The methods were carried out in accordance with the approved guidelines. Written informed consent was obtained from all participants. Families 1, 2 and 4 included one confirmed patient each, and Family 3 included two confirmed patients. The parents of family 1 and family 2 also participated in the study. Clinical data for these subjects was ascertained by detailed ocular examinations, including fundus photograph, multicolor, infrared reflectance (IR), fundus autofluorescence (FAF), fundus fluorescein angiography (FFA)/indocyanine green angiography (ICGA) imaging (HRA2 Heidelberg Engineering, Heidelberg, Germany), optical coherence tomography (OCT; Heidelberg Engineering, Heidelberg, Germany), visual field (VF) (Octopus 900, Switzerland), and full field electroretinography (ffERG; RetiPort ERG system, Roland Consult, Wiesbaden, Germany). Proband II-1 in family 4 is a three-year-child, so we only got the ERG after standard dark adaption under anesthesia. In addition, physical examinations were performed to exclude systemic diseases.

### Mutation analysis

Coding exons of *RDH5, RLBP1*, and *RPE65* were amplified by a polymerase chain reaction (PCR) using primers previously described^24^. The PCR products were sequenced on an ABI3730 Automated Sequencer (PE Biosystems, Foster City, CA). SIFT (http://sift.jcvi.org/)^25^ and Polyphen (http://genetics.bwh.harvard.edu/pph2/)^26^ were used to predict the possible impact of an amino acid substitution on the structure and function of a human protein.

## Additional Information

**How to cite this article:** Yang, G. *et al*. Genetic and phenotypic characteristics of four Chinese families with Fundus Albipunctatus. *Sci. Rep.* **7**, 46285; doi: 10.1038/srep46285 (2017).

**Publisher's note:** Springer Nature remains neutral with regard to jurisdictional claims in published maps and institutional affiliations.

## Figures and Tables

**Table 1:** The clinical features of probands from the four families.

roband Family	Age/Sex	BCVA	Fundus	OCT	IR/FAF	FFA/ICGA	VF
II:1 Family 1	6y/F	0.8/1.0	a, c	Hyperreflective lesions scattering on the periphery of the retina	Normal	Normal angiography	Reduction of sensitivity in the central visual field R MD:3.7 L MD:6.2
II:2 Family 2	21y/M	0.8/0.8	a, c	Hyperreflective lesions scattering on the periphery of the retina	Normal	Normal angiography	No data
II:6 Family 3	41y/F	1.0/0.8	a, b, c	Hyperreflective lesions scattering on the periphery and perimacular area of the retina	Normal	Abnormal in perimacular and periphery	Reduction of sensitivity in the central visual field R MD:11.4 L MD:9.9
II:1 Family 4	3y/M	No data	a, c	No data	No data	No data	No data

Abbreviations: DA. dark adaption; a. white or white-yellow dots in the periphery of the retina; b. white or white-yellow dots in the perimacular area; c. without macular involvement; R.right eye; L.left eye; MD. mean defect.

**Table 2:** The data of ffERG of patients from the four families.

Proband	II:1	II:2	II:6	II:1
Family	Family 1	Family 2	Family 3	Family4
**Mutation**	*RDH5*	*RDH5*	*RDH5*	*RPE65*
	c.928delCinsGAAG+	c.500G > A+	c.928delCinsGAAG+	c.639_640insA+
	c.928delCinsGAAG	c.719ins G	c.500G > A	c. 982C > T
**ffERG**				
Amplitude (uV)				
**Rod** (b-wave)				
** **DA 30 min	0	0	0	0
** **DA 3 h	233	150	84.5	No data
** **DA 10 h	247	173	89.4	No data
**Bright** (b-wave)				
** **DA 30 min	151.0	291.0	44.4	52.9
** **DA 3 h	503.0	457.0	203.0	No data
** **DA 10 h	502.0	454.0	288.0	No data
**Cone** (b-wave)				
** **DA 30 min	161.0	115.0	77.3	30.8
** **DA 3 h	134.0	109.0	37.5	No data
** **DA 10 h	122.0	119.0	65.0	No data
**30-Hz Flicker** (N1-P1)				
** **DA 30 min	147.0	94.5	90.3	16.7
** **DA 3 h	80.5	106.0	73.7	No data
** **DA 10 h	102.0	95.1	89.4	No data

Rod (b-wave): Normal range: b-wave: >80. Bright (b-wave): Normal range: b-wave: 300–380. Cone (b-wave): Normal range: b-wave:100–180. 30-Hz Flicker (N1-P1): Normal range: N1-P1: >80. Abbreviations: ffERG. full field electroretinography.
